# Associations of self-efficacy and perceived benefits and barriers with sugar-sweetened beverage consumption patterns: a secondary analysis of the 2017–2020 Nutrition and Health Survey in Taiwan (NAHSIT)

**DOI:** 10.3389/fnut.2026.1842313

**Published:** 2026-06-30

**Authors:** Chi-Hsuan Liu, Chyi-Huey Bai, Te-Chih Wong, Shu-Chen Lee, Yi-Chun Chen

**Affiliations:** 1 School of Nutrition and Health Sciences, College of Nutrition, Taipei Medical University, Taipei, Taiwan; 2 Department of Public Health, School of Medicine, College of Medicine, Taipei Medical University, Taipei, Taiwan; 3 School of Public Health, College of Public Health, Taipei Medical University, Taipei, Taiwan; 4 Nutrition Research Center, Taipei Medical University Hospital, Taipei, Taiwan; 5 Department of Nutrition and Health Sciences, Chinese Culture University, Taipei, Taiwan; 6 Institute of Biomedical Sciences, Academia Sinica, Taipei, Taiwan

**Keywords:** Nutrition and Health Survey in Taiwan (NAHSIT), perceived barriers, secondary analysis, self-efficacy, sugar-sweetened beverages (SSBs)

## Abstract

**Background:**

Sugar-sweetened beverages (SSBs) contribute to excessive sugar intake and related health risks, with particularly high consumption levels observed in Taiwan. However, limited research has examined psychosocial factors associated with SSB consumption in large population-based surveys. This study examined SSB consumption patterns and their associations with psychosocial factors related to reducing SSB consumption, including self-efficacy, perceived benefits, and perceived barriers.

**Methods:**

Data were obtained from the 2017–2020 Nutrition and Health Survey in Taiwan (NAHSIT). The analytic sample included 4,475 individuals aged 16–64 years, categorized as non-, low-, medium-, or high SSB consumers based on weekly consumption frequency derived from the food frequency questionnaire (FFQ). Psychosocial factors, including self-efficacy (assessed using a perceived difficulty item), perceived benefits, and perceived barriers, were assessed using the “dietary belief” questionnaire. Logistic regression models were used to examine the associations between psychosocial factors and SSB consumption levels.

**Results:**

Most individuals were SSB consumers (88.6%), with 32.1% classified as high consumers (≥1 time/day). Self-efficacy and perceived benefit scores were highest among non- and low consumers, and lowest among high consumers, whereas perceived barrier scores increased with consumption level (*p* < 0.001). In regression analyses, higher self-efficacy was associated with lower odds of SSB consumption, whereas perceived barrier scores were positively associated.

**Conclusion:**

Higher self-efficacy (i.e., lower perceived difficulty) and lower perceived barriers were associated with lower SSB intake. These findings suggest that perceived capability and barriers related to reducing SSB consumption may be relevant factors associated with consumption patterns.

## 1 Introduction

The World Health Organization (WHO) identifies sugar-sweetened beverages (SSBs) as a significant source of excessive sugar intake and a key contributor to increased health risks. Accordingly, the WHO recommends that adults and children limit daily intake of free sugars to less than 10% of total energy, with further reductions to below 5% providing additional health benefits (1). However, global intake of SSBs remains high, exceeding the recommended limits in many high-income countries and continuing to rise in low- and middle-income countries (2). Evidence from several studies suggests that the sugars in SSBs, being in liquid form, are rapidly absorbed and contribute to weight gain (3, 4), which in turn contributes to metabolic abnormalities and elevates the risk of type 2 diabetes, metabolic syndrome, and cardiovascular disease (5–9). These health consequences have emerged as a major public health concern.

Sugar-sweetened beverages (SSBs) are widely consumed in Taiwan, with intake levels among the highest in East Asia (10). According to the 2013–2016 Nutrition and Health Survey in Taiwan (NAHSIT), over 30% of individuals aged 7–44 years consumed SSBs at least once per day (11). This pattern is largely influenced by the widespread availability of SSBs in daily life. As an East Asian country with a strong tea-drinking culture, particularly hand-shaken drinks, SSBs are easily accessible from convenience stores to cafés and tea shops (12). Identifying the factors associated with SSB consumption is essential for developing effective interventions and public health strategies to reduce intake and prevent related health consequences.

While environmental and policy strategies are essential for reducing SSB consumption, individual-level psychosocial factors may also influence beverage choices. Key factors, including self-efficacy, perceived benefits, and perceived barriers, have been shown to play an important role in shaping health-related behaviors (13–16). These constructs are informed by established behavior change theories, including the Social Cognitive Theory and the Health Belief Model (17). Self-efficacy refers to an individual’s confidence in their ability to initiate and maintain behavioral change. In the present study, self-efficacy was assessed using an item reflecting perceived difficulty in reducing SSB consumption. In addition, perceived benefits and perceived barriers may influence individuals’ decisions to adopt healthier behaviors. Together, these psychosocial factors may shape motivation and decision-making related to reducing SSB consumption.

Previous studies on SSB consumption in Taiwan have primarily examined consumption patterns, associated factors, and intervention outcomes (12, 18–20). However, limited research has examined the associations between psychosocial variables and SSB consumption patterns in large population-based surveys. Understanding these relationships may provide insight into the motivational processes underlying beverage choices. Therefore, this study aimed to examine SSB consumption patterns and explore the associations between self-efficacy, perceived benefits, perceived barriers, and different levels of SSB consumption using data from the 2017–2020 NAHSIT. The findings may provide insights for future intervention strategies to reduce SSB intake.

## 2 Materials and methods

### 2.1 Study design and population

This study was conducted in accordance with the STROBE-nut reporting guidelines (21). Data were obtained from the 2017–2020 NAHSIT, a cross-sectional survey based on a multistage, stratified, clustered probability sampling design intended to represent the Taiwanese population (22). NAHSIT employed a complex survey design; however, the present analyses did not apply sampling weights or explicitly account for clustering and stratification. The primary aim of this study was to examine associations between psychosocial factors and SSB consumption. Detailed descriptions of the survey design, data collection, and analytic methods have been reported elsewhere (22, 23).

**Figure 1:**
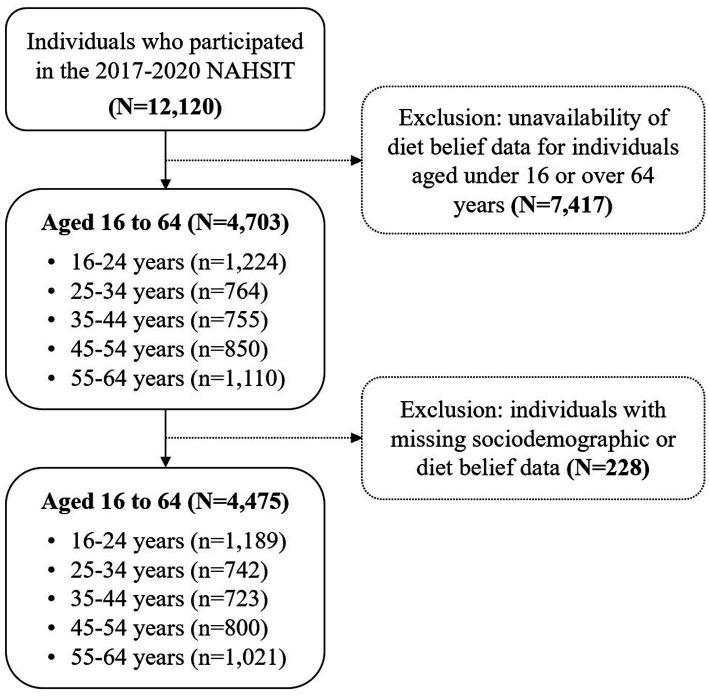
Flow chart of the study sample selection process.

For the present analysis, individuals younger than 16 years or older than 64 years (*n* = 7,417) were excluded because dietary belief data were not available for these age groups. Individuals with missing sociodemographic or dietary belief data were also excluded (*n* = 228) rather than imputed to avoid introducing bias from estimated values, resulting in a final analytic sample of 4,475 (Figure 1). The original NAHSIT study was conducted in accordance with the Declaration of Helsinki and approved by the Institutional Review Board of Biomedical Science Research, Academia Sinica (Project number: AS-IRB-BM-16066; Approval date: 7 March 2017). The present secondary analysis was reviewed and approved by the Taipei Medical University-Joint Institutional Review Board (TMU-JIRB; No. N202503016).

### 2.2 Study variables

#### 2.2.1 SSB consumption

In the 2017–2020 NAHSIT, SSB consumption data were obtained from the Food Frequency Questionnaire (FFQ) (22), in which SSBs were defined as non-alcoholic beverages with added sugar, including coffee and tea, carbonated soft drinks, fruit drinks, sports and energy drinks, and non-caffeinated beverages. Beverages without added sugar (e.g., unsweetened tea and coffee, and other sugar-free beverages) were classified as non-SSBs and were not included. Participants reported their consumption frequency over the past month (daily, weekly, or monthly), and all responses were converted into estimated weekly frequencies.

To derive an overall SSB consumption frequency, the reported frequencies for each SSB item were summed to obtain a total weekly frequency (times/week). All listed SSB categories in the FFQ were included in this calculation, as they represent the major sources of added sugar-containing beverages in the Taiwanese diet. For participants who reported consuming multiple SSB types, the total weekly frequency reflected the combined consumption across all SSB categories. This approach assumes that each reported consumption occasion contributes equally to overall SSB intake frequency.

Individuals were categorized into four groups based on the distribution of consumption frequencies to facilitate group comparisons and enhance the interpretability of consumption patterns. Non-consumers were defined as those reporting no consumption (0 times/week). The remaining participants were classified into tertiles as low consumers (1–2 times/week), medium consumers (3–6 times/week), and high consumers (≥7 times/week, equivalent to ≥1 time/day), following the approach used in a previous study (24).

#### 2.2.2 Psychosocial factors related to reducing SSB consumption

Psychosocial factors were derived from SSB-related items in the “dietary belief” questionnaire of NAHSIT (22), assessing self-efficacy, perceived benefits, and perceived barriers related to reducing SSB consumption. All responses were measured on a five-point Likert scale ranging from 1 (strongly agree) to 5 (strongly disagree). The measures included:

(1) A single-item indicator reflecting perceived difficulty in reducing SSB consumption, used as a proxy for self-efficacy (score range: 1–5). Higher scores indicate lower perceived difficulty (i.e., greater inferred self-efficacy).
(2) A four-item perceived benefits scale assessing the perceived advantages of reducing SSB consumption (score range: 4–20). All items were reverse-coded so that higher scores indicate stronger perceived benefits.
(3) A four-item perceived barriers scale assessing the perceived obstacles to reducing SSB consumption (score range: 4–20). All items were reverse-coded so that higher scores indicate stronger perceived barriers.

The questionnaire items are presented in Table 1. Internal consistency reliability was evaluated for the multi-item scales using Cronbach’s alpha, with coefficients of 0.780 for perceived benefits and 0.699 for perceived barriers. Reliability was not assessed for the single-item self-efficacy measure.

**Table 1:** Items of psychosocial scales related to reducing sugar-sweetened beverage (SSB) consumption from the NAHSIT “dietary belief” questionnaire.

Scale	Item
Self-efficacy *Single item; score range: 1–5*	For me, reducing SSB consumption is very difficult
Perceived benefits *4 items; score range: 4–20*	Reducing SSB consumption can lower the risk of developing dental cavities Reducing SSB consumption can help with weight loss or maintaining a healthy weight Reducing SSB consumption makes my body feel lighter and less physically burdened Overall, reducing SSB consumption is very beneficial to me
Perceived barriers *4- items; score range: 4–20*	I like having SSBs with my meals I enjoy the taste of SSBs When I am with classmates, friends, relatives, or colleagues, I find it difficult to avoid consuming SSBs When I am hungry, I often drink SSBs to boost my energy

Responses were assessed using a five-point Likert scale (1 = strongly agree to 5 = strongly disagree). The self-efficacy measure was based on a perceived difficulty item, with higher scores indicating lower perceived difficulty (i.e., higher self-efficacy). All items for perceived benefits and perceived barriers were also reverse-coded, so that higher scores indicate greater perceived benefits and higher perceived barriers.

#### 2.2.3 Sociodemographic characteristics

Sociodemographic characteristics included sex (male, female), age group (16–24, 25–34, 35–44, 45–54, and 55–64 years), education level (high school or below, college or higher), perceived health status (better than others, about the same as others, worse than others), and BMI (underweight, normal weight, overweight/obese). BMI was calculated using height and weight measured by trained interviewers and classified according to the standards established by the Ministry of Health and Welfare in Taiwan (25, 26). These variables were obtained from the sociodemographic questionnaire of the 2017–2020 NAHSIT (22) and were selected as potential covariates based on previously reported associations with SSB consumption (27, 28).

### 2.3 Statistical analysis

All analyses were conducted using SPSS version 26.0 (IBM Corp., Armonk, N.Y., USA), with statistical significance set at *p* < 0.05. Descriptive statistics were used to summarize sample characteristics, with categorical variables presented as frequencies and percentages, and continuous variables as means ± standard deviations.

Chi-squared tests were used to examine differences in sociodemographic characteristics across SSB consumer groups. One-way analysis of variance (ANOVA), followed by Scheffe’s post-hoc test, was conducted to compare mean scores of self-efficacy, perceived benefits, and perceived barriers across SSB consumer groups. For descriptive analyses, responses to individual Likert-scale items were collapsed into three categories (agree, neither agree nor disagree, and disagree), with “strongly agree” combined with “agree” and “strongly disagree” combined with “disagree,” to improve interpretability and ensure adequate cell counts for chi-square tests.

Logistic regression models were used to examine the associations between psychosocial variables and SSB consumption, adjusting for sociodemographic covariates. Multicollinearity among psychosocial variables was assessed using variance inflation factors (VIFs). No evidence of multicollinearity was observed, with VIF values ranging from 1.06 to 1.78. Three models were specified: (1) Model 1, a binary logistic regression comparing SSB consumers with non-consumers; (2) Model 2, a multinomial logistic regression including all four consumer groups, with non-consumers as the reference group; and (3) Model 3, a multinomial logistic regression excluding non-consumers, with low consumers as the reference group. Model fit was assessed using likelihood ratio tests, and related model-fitting information is reported in the table footnotes.

Sensitivity analyses were conducted by treating SSB consumption as a continuous variable to assess the robustness of the findings to different specifications of the outcome variable.

To explore potential age-specific associations, stratified analyses were conducted by age group (16–24, 25–34, 35–44, 45–54, and 55–64 years). Within each age group, the same three regression models were fitted with the same covariate adjustments as in the main analyses.

These model specifications, along with stratified and sensitivity analyses, allowed assessment of the consistency of findings across different outcome definitions and population subgroups.

## 3 Results

### 3.1 Sample characteristics across SSB consumer groups

A total of 4,475 individuals were included in this analysis. The characteristics of the sample across SSB consumer groups are summarized in Table 2. The mean (SD) age of the samples was 38.6 (15.8) years, and 50.3% of them were female (*n* = 2,252). More than 60% had a high school education or lower (63.8%, *n* = 2,854), 49.6% perceived their health status as about the same as others (*n* = 2,219), and 47.7% were overweight/obese (*n* = 2,133). Overall, the mean SSB intake was 5.5 times/week. Nearly 90% were SSB consumers (88.6%, *n* = 3,965), with 32.1% as high consumers (≥1 time/day, *n* = 1,437).

**Table 2:** Sociodemographic characteristics of the analytic sample by SSB consumer groups (NAHSIT 2017–2020, *N* = 4,475).

Characteristics	*n*	SSB consumer groups, %				*p*-value^1^
		Non-consumers (0 times/week)	Low consumers (1–2 times/week)	Medium consumers (3–6 times/week)	High consumers (≥1 time/day)	
	4,475	11.4	28.9	27.6	32.1	
Sex						**< 0.001** ^***^
Male	2,223	9.9	25.3	27.4	37.4	
Female	2,252	12.9	32.5	27.7	26.9	
Age (years)						**< 0.001** ^***^
16–24	1,189	3.3	20.6	35.4	40.7	
25–34	742	5.9	25.1	26.3	42.7	
35–44	723	8.0	26.3	29.0	36.7	
45–54	800	14.4	36.0	24.4	25.2	
55–64	1,021	24.9	37.8	20.8	16.5	
Education level						**< 0.001** ^***^
High school or below	2,854	11.6	26.9	26.8	34.7	
College or higher	1,621	11.1	32.4	28.9	27.6	
Perceived health status						**< 0.001** ^***^
Worse than others	1,092	9.7	27.7	28.2	34.4	
About the same as others	2,219	10.2	28.6	27.4	33.8	
Better than others	1,164	15.2	30.9	27.3	26.6	
BMI						**0.005** ^**^
Underweight	266	8.3	20.7	31.2	39.8	
Normal weight	2,076	11.2	30.5	26.7	31.6	
Overweight/Obese	2,133	12.0	28.5	27.9	31.6	

SSB, sugar-sweetened beverage; BMI, body mass index. ^*^p < 0.05, ^**^p < 0.01, ^***^p < 0.001.

1 Chi-squared tests.

Bold values indicate statistical significance.

The prevalence of SSB consumption was highest among individuals aged 16–24 years (96.7%), followed by those aged 25–34 years (94.1%), 35–44 years (92.0%), 45–54 years (85.6%), and 55–64 years (75.1%). High consumers were more prevalent among males (37.4%), younger individuals aged 16–44 years (36.7–42.7%), and those with a high school education or below (34.7%; all *p* < 0.001). Higher proportions were also observed among individuals who perceived their health status as being about the same as others (33.8%) or worse than others (34.4%), as well as among those classified as underweight (39.8%; all *p* < 0.001). Conversely, non-consumers were more prevalent among females (12.9%), individuals aged 45–54 (14.4%) or 55–64 (24.9%) years, and those perceiving their health status as better than others (15.2%; Table 2).

### 3.2 Psychosocial factors across SSB consumer groups

Mean scores of self-efficacy, perceived benefits, and perceived barriers across SSB consumer groups are presented in Table 3. Higher self-efficacy (i.e., lower perceived difficulty) scores were observed among non- and low consumers (4.14 ± 0.57 and 4.04 ± 0.66, respectively) and were lowest among high consumers (3.15 ± 1.10; *p* < 0.001). Similarly, perceived benefit scores decreased significantly with consumption level, whereas perceived barrier scores increased, ranging from 8.36 ± 2.15 in non-consumers to 12.18 ± 2.98 in high consumers (all *p* < 0.001).

**Table 3:** Mean scores of psychosocial factors by SSB consumer groups^1^.

Variables	SSB consumer groups				*p*-value^2^
	Non-consumers (0 times/week)	Low consumers (1–2 times/week)	Medium consumers (3–6 times/week)	High consumers (≥1 time/day)	
Self-efficacy	4.14 ± 0.57^a^	4.04 ± 0.66^a^	3.64 ± 0.89^b^	3.15 ± 1.10^c^	**< 0.001** ^**###**^
Perceived benefits	16.26 ± 2.07^a^	16.21 ± 2.06^a^	15.85 ± 2.15^b^	15.50 ± 2.40^c^	**< 0.001** ^**###**^
Perceived barriers	8.36 ± 2.15^d^	9.22 ± 2.36^c^	10.63 ± 2.56^b^	12.18 ± 2.98^a^	**< 0.001** ^**###**^

SSB, sugar-sweetened beverage. ^#^*p* < 0.05, ^##^*p* < 0.01, ^###^*p* < 0.001. ^a,b,c,d^Means within each row sharing the same letter are not significantly different at the 5% level according to one-way ANOVA with Scheffe’s *post-hoc* test.

1 Data are presented as mean ± standard deviation.

2 One-way ANOVA.

Bold values indicate statistical significance.

The distribution of responses to psychosocial items across SSB consumer groups is presented in Table 4, and the patterns were generally consistent with the trends observed in Table 3. Agreement with the statement that “*reducing SSB consumption is very difficult*” increased with consumption level, from 2.2% in non-consumers to 35.5% in high consumers. For perceived benefit items, agreement was generally high across all groups, although relatively lower agreement (approximately 70–80%) was observed for items related to “*dental health*” and “*perceived physical burden*.” In contrast, agreement with perceived barrier items increased with consumption level, particularly for items related to social contexts and taste preference. Difficulty avoiding SSBs in “*social situations*” showed a marked increase across groups (24.3% in non-consumers to 55.4% in high consumers), and agreement with “*enjoying the taste of SSBs*” was also higher among those with greater consumption levels (Table 4).

**Table 4:** Distribution of responses to psychosocial items by SSB consumer groups.

	Total	Non-consumers (0 times/week)	Low consumers (1–2 times/week)	Medium consumers (3–6 times/week)	High consumers (≥1 time/day)	*p*-value^1^
Items	4,475 (100.0)	510 (100.0)	1,295 (100.0)	1,233 (100.0)	1,437 (100.0)	
Self-efficacy						
For me, reducing SSB consumption is very difficult						**<0.001** ^***^
Agree	784 (17.5)	11 (2.2)	59 (4.6)	204 (16.5)	510 (35.5)	
Neither agree nor disagree	356 (8.0)	16 (3.1)	61 (4.7)	121 (9.8)	158 (11.0)	
Disagree	3,335 (74.5)	483 (94.7)	1,175 (90.7)	908 (73.6)	769 (53.5)	
Perceived benefits						
Reducing SSB consumption can lower the risk of developing dental cavities						**<0.001** ^***^
Agree	3,762 (84.1)	455 (89.2)	1,134 (87.6)	1,031 (83.6)	1,142 (79.5)	
Neither agree nor disagree	406 (9.1)	28 (5.5)	77 (5.9)	119 (9.7)	182 (12.7)	
Disagree	307 (6.9)	27 (5.3)	84 (6.5)	83 (6.7)	113 (7.9)	
Reducing SSB consumption can help with weight loss or maintaining a healthy weight						**<0.001** ^***^
Agree	4,019 (89.8)	476 (93.3)	1,208 (93.3)	1,117 (90.6)	1,218 (84.8)	
Neither agree nor disagree	268 (6.0)	18 (3.5)	53 (4.1)	63 (5.1)	134 (9.3)	
Disagree	188 (4.2)	16 (3.1)	34 (2.6)	53 (4.3)	85 (5.9)	
Reducing SSB consumption makes my body feel lighter and less physically burdened						**<0.001** ^***^
Agree	3,498 (78.2)	460 (90.2)	1,106 (85.4)	946 (76.7)	986 (68.6)	
Neither agree nor disagree	650 (14.5)	29 (5.7)	123 (9.5)	199 (16.1)	299 (20.8)	
Disagree	327 (7.3)	21 (4.1)	66 (5.1)	88 (7.1)	152 (10.6)	
Overall, reducing SSB consumption is very beneficial to me						**<0.001** ^***^
Agree	3,939 (87.8)	468 (91.8)	1,182 (91.3)	1,094 (88.7)	1,185 (82.5)	
Neither agree nor disagree	359 (8.0)	25 (4.9)	74 (5.7)	91 (7.4)	169 (11.8)	
Disagree	187 (4.2)	17 (3.3)	39 (3.0)	48 (3.9)	83 (5.8)	
Perceived barriers						
I like having SSBs with my meals						**<0.001** ^***^
Agree	912 (20.4)	10 (2.0)	88 (6.8)	237 (19.2)	577 (40.2)	
Neither agree nor disagree	405 (9.1)	17 (3.3)	71 (5.5)	139 (11.3)	178 (12.4)	
Disagree	3,158 (70.6)	483 (94.7)	1,136 (87.7)	857 (69.5)	682 (47.5)	
I enjoy the taste of SSBs						**<0.001** ^***^
Agree	1,364 (30.5)	41 (8.0)	200 (15.4)	392 (31.8)	731 (50.9)	
Neither agree nor disagree	665 (14.9)	27 (5.3)	155 (12.0)	228 (18.5)	255 (17.7)	
Disagree	2,446 (54.7)	442 (86.7)	940 (72.6)	613 (49.7)	451 (31.4)	
When I am with classmates, friends, relatives, or colleagues, I find it difficult to avoid consuming SSBs						**<0.001** ^***^
Agree	1,876 (41.9)	124 (24.3)	413 (31.9)	543 (44.0)	796 (55.4)	
Neither agree nor disagree	384 (8.6)	17 (3.3)	92 (7.1)	129 (10.5)	146 (10.2)	
Disagree	2,215 (49.5)	369 (72.4)	790 (61.0)	561 (45.5)	495 (34.4)	
Perceived barriers						
When I am hungry, I often drink SSBs to boost my energy						**<0.001** ^***^
Agree	956 (21.4)	38 (7.5)	174 (13.4)	258 (20.9)	486 (33.8)	
Neither agree nor disagree	263 (5.9)	23 (4.5)	53 (4.1)	86 (7.0)	101 (7.0)	
Disagree	3,256 (72.8)	449 (88.0)	1,068 (82.5)	889 (72.1)	850 (59.2)	

SSB, sugar-sweetened beverage. Responses were originally measured on a 5-point Likert scale and collapsed into three categories for presentation. ^*^p < 0.05, ^**^p < 0.01, ^***^p < 0.001.

1 Chi-square tests. Bold values indicate statistical significance.

### 3.3 Logistic regression analysis of psychosocial factors associated with SSB consumption

Table 5 summarizes the results of the binary and multinomial logistic regression models examining the association between psychosocial factors and SSB consumption, adjusted for sociodemographic variables, with non-consumers as the reference group. In Model 1, higher self-efficacy (i.e., lower perceived difficulty) was associated with lower odds of being an SSB consumer (OR = 0.75, *p* = 0.005), whereas higher perceived barrier scores were associated with higher odds (OR = 1.29, *p* < 0.001). In Model 2, compared with non-consumers, higher self-efficacy was associated with lower odds of being a medium consumer (OR = 0.70, *p* = 0.001) or a high consumer (OR = 0.57, *p* < 0.001). In contrast, higher perceived barrier scores were associated with higher odds of being a low (OR = 1.18, *p* < 0.001), medium (OR = 1.33, *p* < 0.001), or high consumer (OR = 1.55, *p* < 0.001).

**Table 5:** Logistic regression analyses of psychosocial factors associated with SSB consumption (reference: non-consumers)^1^.

Psychosocial factors	Model 1^†^			Model 2^‡^								
	Consumers vs. Non-consumers			Low vs. Non			Medium vs. Non			High vs. Non		
	OR	95% CI	*p* value	OR	95% CI	*p* value	OR	95% CI	*p* value	OR	95% CI	*p* value
Self-efficacy	**0.75**	**0.61–0.92**	**0.005** ^**^	1.01	0.81–1.26	0.945	**0.70**	**0.56–0.87**	**0.001** ^**^	**0.57**	**0.46–0.70**	**<0.001** ^***^
Perceived benefits	0.99	0.94–1.04	0.669	1.00	0.94–1.06	0.935	0.97	0.92–1.03	0.279	0.94	0.89–1.00	0.053
Perceived barriers	**1.29**	**1.23–1.37**	**<0.001** ^***^	**1.18**	**1.11–1.25**	**<0.001** ^***^	**1.33**	**1.26–1.42**	**<0.001** ^***^	**1.55**	**1.45–1.65**	**<0.001** ^***^

Independent variables: self-efficacy, perceived benefits, and perceived barriers. Dependent variable: SSB consumer group. Models were adjusted for sex, age, education level, perceived health, and body mass index (BMI). SSB, sugar-sweetened beverage; OR, odds ratio; CI, confidence interval. Significance levels: ^*^p < 0.05, ^**^p < 0.01, ^***^p < 0.001. ^†^Model 1: Binary logistic regression comparing SSB consumers (*n* = 3,965) vs. non-consumers (*n* = 510). Model fitting information: *p* < 0.001. ^‡^Model 2: Multinomial logistic regression including all four consumer groups (*N* = 4,475), using non-consumers as the reference group. Model fitting information: *p* < 0.001.

1 SSB consumer groups were classified based on intake distribution, with non-consumers as the first group (0 times/week), and the remaining consumers divided into tertiles: low consumers (1–2 times/week), medium consumers (3–6 times/week), and high consumers (≥1 time/day). Bold values indicate statistical significance.

The results of Model 3 are presented in Table 6. Similar to Model 2, compared with low consumers, higher self-efficacy was associated with lower odds of being a medium consumer (OR = 0.70, *p* < 0.001) or a high consumer (OR = 0.56, *p* < 0.001). In contrast, higher perceived barrier scores were associated with higher odds of being a medium (OR = 1.14, *p* < 0.001) or high consumer (OR = 1.32, *p* < 0.001). Additionally, higher perceived benefit scores were associated with lower odds of being a high consumer (OR = 0.94, *p* = 0.007), although this association was not observed for medium consumers.

**Table 6:** Multinomial logistic regression analysis of psychosocial factors associated with SSB consumption among consumers (reference: low consumers, *N* = 3,965)^1^.

Psychosocial factors	Medium vs. Low			High vs. Low		
	OR	95% CI	*p* value	OR	95% CI	*p* value
Self-efficacy	**0.70**	**0.61–0.79**	**<0.001** ^***^	**0.56**	**0.50–0.64**	**<0.001** ^***^
Perceived benefits	0.97	0.93–1.01	0.141	**0.94**	**0.91–0.99**	**0.007** ^**^
Perceived barriers	**1.14**	**1.09–1.18**	**<0.001** ^***^	**1.32**	**1.27–1.38**	**<0.001** ^***^

Independent variables: self-efficacy, perceived benefits, and perceived barriers. Dependent variable: SSB consumer group (excluding non-consumers), with low consumers as the reference group. The model was adjusted for sex, age, education level, perceived health, and body mass index (BMI). Model fitting information: *p* < 0.001. SSB, sugar-sweetened beverage; OR, odds ratio; CI, confidence interval. Significance levels: ^*^p < 0.05, ^**^p < 0.01, ^***^p < 0.001.

1 SSB consumer groups were divided into tertiles based on intake distribution: low consumers (1–2 times/week), medium consumers (3–6 times/week), and high consumers (≥1 time/day). Bold values indicate statistical significance.

Sensitivity analysis treating SSB consumption as a continuous variable showed that higher self-efficacy and higher perceived benefits were significantly associated with lower overall SSB consumption. In contrast, higher perceived barriers were positively associated with SSB consumption (Supplementary Table S1).

Stratified analyses by age group are presented in Supplementary Table S2. Overall, higher perceived barrier scores were consistently associated with greater SSB consumption across models in all age groups, except those aged 25–34 years. In this age group, self-efficacy showed the strongest association with SSB consumption across Models 1, 2, and 3. In contrast, for the other age groups, self-efficacy was not significantly associated with consumption in Models 1 or 2 but was inversely associated in Model 3. Additionally, among those aged 55–64 years, higher perceived benefit scores were associated with lower odds of being medium consumers compared with low consumers in Model 3 (OR = 0.91, *p* = 0.046; Supplementary Table S2).

## 4 Discussion

This study examined SSB consumption patterns among individuals aged 16 to 64 years and explored the associations between self-efficacy (assessed using a perceived difficulty item), perceived benefits, perceived barriers, and SSB consumption levels using data from the 2017–2020 NAHSIT. The findings indicate that SSB consumption is highly prevalent in Taiwan, with 88.6% of individuals reporting consumption and more than 30% consuming SSBs at least once per day. Psychosocial factors, particularly self-efficacy (i.e., perceived difficulty) and perceived barriers, were important correlates of SSB consumption behaviors. To our knowledge, this study represents one of the first comprehensive analyses in Asia to examine psychosocial factors related to reducing SSB consumption using data from a large population-based survey.

### 4.1 SSB consumption patterns and characteristics among high consumers

In this study, the mean SSB intake was 5.5 times/week, and 32.1% of individuals were classified as high consumers (≥1 time/day). Previous research in Taiwan has consistently shown that sweetened tea beverages are the most commonly consumed form of SSBs and account for a substantial proportion of total sugar intake, particularly among younger populations (12, 29, 30). Therefore, the observed SSB consumption patterns in this study are likely to reflect the intake of sweetened tea and hand-shaken beverages. According to a 2023 study on hand-shaken tea drinks in Taiwan, the average sugar content of a regular-sugar tea was 51.1 g per cup (31). Assuming a daily energy intake of 2000 kcal, consumption of a single cup would already exceed 10% of the total energy intake from added sugars, which is the upper limit recommended by both the WHO and the Taiwan Dietary Guidelines (1, 32). These findings highlight the substantial public health burden of SSB consumption in Taiwan.

Furthermore, we found that SSB consumption patterns varied significantly across sociodemographic groups. Males and individuals younger than 44 years were more likely to be high consumers, whereas females and older adults tended to limit their intake. Notably, the prevalence of SSB consumers was highest among individuals aged 16–24 years, whereas the proportion of high consumers peaked in those aged 25–34 years (42.7%), indicating distinct age-related consumption patterns. These findings align with previous NAHSIT data and other Taiwanese studies reporting higher SSB consumption among males and younger populations (11, 33–35).

Consistent with global evidence, younger individuals and males appear to have higher SSB consumption and may represent key groups for intervention. Previous studies across different regions have shown higher SSB consumption among males and younger populations, despite variations in overall intake levels (10, 36–38). These findings suggest that, regardless of national differences in consumption patterns, these groups may be important targets for intervention. In addition, individuals with lower education levels were more likely to be high consumers, consistent with findings from studies conducted in the United States and Canada (39, 40). This pattern may be related to underlying socioeconomic disparities in health behaviors and underscores the need for targeted interventions among vulnerable populations.

### 4.2 Associations and potential roles of psychosocial factors in SSB consumption

This study found that high consumers had the lowest mean scores for self-efficacy (i.e., the greatest perceived difficulty) and perceived benefits, and the highest mean score for perceived barriers. Lower self-efficacy among high consumers suggests that individuals with greater intake may report lower confidence in their ability to reduce SSB consumption, consistent with previous studies (41–43). Similar patterns have also been observed in other health behaviors, where individuals with less healthy behaviors tend to exhibit lower self-efficacy, lower perceived benefits, and higher perceived barriers (44–46).

The item-level analysis further supports these findings. Although perceived benefits of reducing SSB consumption were generally high across all groups, agreement was relatively lower for specific aspects, such as dental health and perceived physical burden. This may indicate that general awareness of health benefits is not necessarily associated with lower SSB consumption when specific risks are less salient. Previous research suggests that risk perception is more likely to increase with direct experience or exposure to prominent cues, such as media coverage or personal encounters (47).

In contrast, perceived barrier scores were positively associated with SSB consumption levels. Social contexts and taste preferences showed relatively high levels of agreement across all consumer groups, indicating that these barriers are pervasive rather than limited to high consumers. These findings indicate that environmental and hedonic factors may be relevant to SSB consumption behaviors (41, 48). Specifically, difficulty avoiding SSBs in social situations may be associated with social norms and peer contexts (49, 50), while strong taste preferences may be associated with habitual consumption patterns (51, 52). In the Taiwanese context, these barriers may be related to the widespread availability and social role of hand-shaken drinks, which emphasize taste customization and social interaction (31).

Regression analyses further clarified the relative importance of psychosocial factors. Across all models, self-efficacy (perceived difficulty) and perceived barriers emerged as the most consistent correlates of SSB consumption, whereas perceived benefits were not significantly associated. Higher self-efficacy was associated with lower odds of being a medium or high consumer, while higher perceived barriers were associated with greater SSB consumption. These findings suggest that behavioral constraints may be more strongly associated with SSB consumption than perceived benefits (53).

Stratified analyses further revealed age-specific differences. Among the 25–34 age group, self-efficacy showed the strongest association with SSB consumption, whereas perceived barriers were more consistently associated with consumption in other age groups. This pattern suggests that different psychosocial variables may be more relevant across age groups. Addressing perceived barriers (e.g., availability and taste satisfaction) (18, 54) may be particularly relevant in some groups, while perceived difficulty (proxy for self-efficacy) may be more relevant in others.

When considered alongside consumption patterns, younger populations may face greater challenges in reducing SSB intake, which may be related to lower perceived susceptibility to long-term health risks (41, 55) and stronger environmental influences (31, 56). Overall, these findings suggest that psychosocial factors may be relevant to understanding SSB consumption and may provide insights for the development of age-specific intervention strategies.

### 4.3 Implication for public health strategies

These findings may have important implications for public health strategies aimed at reducing SSB consumption. Interventions may consider targeting perceived difficulty (as a proxy for self-efficacy) and addressing perceived barriers, particularly those related to taste preferences and social contexts. Practical approaches may include promoting gradual sugar reduction, providing appealing low-sugar alternatives, and supporting individuals in managing SSB consumption in social situations (57). While this study focused on individual-level factors, SSB consumption occurs within broader environmental contexts. In Taiwan, the widespread availability and social integration of hand-shaken drinks may be associated with these barriers. Therefore, environment-level strategies, such as marketing and labeling policies, price interventions, and improving access to healthier beverage options, may complement individual-level interventions (58). Together, these findings suggest that both individual and environmental factors may be relevant to SSB consumption and may provide insights for the development of integrated strategies.

### 4.4 Strengths and limitations

This study provides novel evidence on SSB consumption and related psychosocial factors among individuals aged 16–64 in Taiwan. A key strength is the use of data from the 2017–2020 NAHSIT, which provides a large, well-characterized population-based sample.

Nevertheless, several limitations should be acknowledged. First, the use of secondary, self-reported data may introduce recall bias and misreporting, potentially leading to underestimation of SSB consumption. Second, the self-efficacy measure in this study was based on a single item reflecting perceived difficulty in reducing SSB consumption and was used as a proxy indicator of self-efficacy; this measure may also capture related constructs such as perceived barriers and does not fully align with the classical definition of self-efficacy. Therefore, the findings should be interpreted with caution. Although multicollinearity diagnostics indicated minimal statistical overlap between these constructs (VIFs = 1.06–1.78), they may still reflect conceptually related aspects of behavioral perception. Third, household income was excluded from the analysis due to substantial missing data, limiting the ability to fully account for socioeconomic factors and potentially leading to residual confounding related to financial capacity. Fourth, although NAHSIT is a nationally representative survey using a complex sampling design, the present analyses did not incorporate sampling weights or account for clustering and stratification. This may affect the precision of standard errors and limit the generalizability of the findings to the national population. Accordingly, the findings should be interpreted as associations within the analytic sample. However, the overall patterns were generally consistent across model specifications, and similar associations were observed across most age groups, which may provide some support for the consistency of the findings despite potential limitations related to sampling design. Finally, given the cross-sectional design of this study, causal relationships between SSB consumption and psychosocial factors cannot be inferred, nor can long-term behavioral changes be assessed. In addition, the observed associations may be subject to reverse causality, and SSB consumption may in turn shape these psychosocial factors.

These strengths and limitations should be considered when interpreting the findings. Future longitudinal and intervention studies may help to further examine the relationships between psychosocial factors and SSB consumption and to incorporate more comprehensive social, environmental, and cultural influences on beverage consumption behaviors.

## 5 Conclusion

Based on the analyses of the 2017–2020 NAHSIT data, this study identified distinct patterns of SSB consumption in relation to psychosocial factors among Taiwanese populations. Adolescents and young adults were more likely to be high consumers, suggesting that younger populations may be an important group for targeted interventions. Higher self-efficacy (i.e., lower perceived difficulty) and lower perceived barriers were associated with lower SSB intake, whereas perceived benefits alone were not clearly associated with consumption. Item-level findings further suggest that taste preferences and social factors are associated with higher SSB consumption. Overall, these findings highlight psychosocial factors associated with SSB intake and provide insights for the development of targeted public health strategies.

## Data Availability

The datasets presented in this article are not publicly available due to legal restrictions on personal health data in Taiwan. Access to the datasets requires formal application and approval from the relevant authority. Information on data application procedures can be found at: https://www.hpa.gov.tw/.
