# Supplementary material for: Associations of self-efficacy and perceived benefits and barriers with sugar-sweetened beverage consumption patterns: a secondary analysis of the 2017–2020 Nutrition and Health Survey in Taiwan (NAHSIT)
Source: Front Nutr. 2026 Jun 30;13:1842313. doi: 10.3389/fnut.2026.1842313 (PMC13364910; doi:10.3389/fnut.2026.1842313)
Supplement: Supplementary file 1 [file Table_1.docx]

Supplementary Material

**Table S1.** Multiple linear regression analysis of sugar-sweetened beverage (SSB) consumption treated as a continuous variable.

|  | **B** | **95% CI** | ***p* value** |
| --- | --- | --- | --- |
| **Self-efficacy** | -1.33 | [-1.58, –1.08] | <0.001^***^ |
| **Perceived benefits** | -0.09 | [-0.18, –0.01] | 0.033^*^ |
| **Perceived barriers** | 0.57 | [0.49, 0.65] | <0.001^***^ |

Independent variables: self-efficacy, perceived benefits, and perceived barriers. Dependent variable: total sugar-sweetened beverage (SSB) consumption (times/week).

The model was adjusted for sex, age, education level, perceived health, and body mass index (BMI). B, unstandardized coefficient; CI, confidence interval.

Significance levels: ^*^*p* < 0.05, ^**^*p* < 0.01, ^***^*p* < 0.001.

**Table S2.** Logistic regression analyses of psychosocial factors associated with SSB consumption by age group: binary and multinomial models ^1^.

|  | **Model 1: binary ^†^** |  | **Model 2: multinomial (4 groups) ^‡^** | | | | |  | **Model 3: multinomial (3 groups) ^§^** | | |
| --- | --- | --- | --- | --- | --- | --- | --- | --- | --- | --- | --- |
|  | **Consumers vs.**  **Non-consumers** |  | **Low vs. Non** |  | **Medium vs. Non** |  | **High vs. Non** |  | **Medium vs. Low** |  | **High vs. Low** |
|  | **OR (95% CI)** |  | **OR (95% CI)** |  | **OR (95% CI)** |  | **OR (95% CI)** |  | **OR (95% CI)** |  | **OR (95% CI)** |
| **16-24 y *(n=1,189)*** |  |  |  |  |  |  |  |  |  |  |  |
| **Self-efficacy** | 1.00 (0.64–1.56) |  | 1.37 (0.85–2.20) |  | **0.99 (0.63–1.56)** |  | **0.79 (0.50–1.25)** |  | **0.72 (0.58–0.91)^**^** |  | **0.58 (0.46–0.73)^***^** |
| **Perceived benefits** | 0.91 (0.77–1.06) |  | 0.91 (0.77–1.07) |  | 0.91 (0.77–1.07) |  | 0.87 (0.73–1.02) |  | 1.00 (0.93–1.08) |  | 0.95 (0.88–1.03) |
| **Perceived barriers** | **1.37 (1.19–1.59)^***^** |  | **1.21 (1.04–1.40)^*^** |  | **1.41 (1.21–1.64)^***^** |  | **1.63 (1.40–1.91)^***^** |  | **1.17 (1.08–1.26)^***^** |  | **1.36 (1.25–1.47)^***^** |
| **25-34 y *(n=742)*** |  |  |  |  |  |  |  |  |  |  |  |
| **Self-efficacy** | **0.44 (0.25–0.77)^**^** |  | 0.65 (0.35–1.20) |  | **0.42 (0.23–0.78)^**^** |  | **0.32 (0.17–0.58)^***^** |  | **0.64 (0.47–0.87)^**^** |  | **0.48 (0.36–0.64)^***^** |
| **Perceived benefits** | 0.98 (0.84–1.14) |  | 1.01 (0.86–1.18) |  | 0.95 (0.81–1.12) |  | 0.95 (0.80–1.11) |  | 0.95 (0.86–1.04) |  | 0.94 (0.85–1.03) |
| **Perceived barriers** | 1.07 (0.93–1.24) |  | 1.00 (0.86–1.17) |  | 1.07 (0.92–1.25) |  | **1.23 (1.06–1.44)^**^** |  | 1.07 (0.97–1.18) |  | **1.23 (1.12–1.35)^***^** |
| **35-44 y *(n=723)*** |  |  |  |  |  |  |  |  |  |  |  |
| **Self-efficacy** | **0.44 (0.25–0.77)^**^** |  | 0.65 (0.35–1.20) |  | **0.42 (0.23–0.78)^**^** |  | **0.32 (0.17–0.58)^***^** |  | **0.68 (0.49–0.92)^*^** |  | **0.58 (0.43–0.79)^***^** |
| **Perceived benefits** | 0.98 (0.84–1.14) |  | 1.01 (0.86–1.18) |  | 0.95 (0.81–1.12) |  | 0.95 (0.80–1.11) |  | 1.03 (0.93–1.13) |  | 0.93 (0.84–1.03) |
| **Perceived barriers** | 1.07 (0.93–1.24) |  | 1.00 (0.86–1.17) |  | 1.07 (0.92–1.25) |  | **1.23 (1.06–1.44)^**^** |  | **1.15 (1.04–1.27)^**^** |  | **1.38 (1.24–1.52)^***^** |

**Table S2.** *Cont.*

|  | **Model 1: binary ^†^** |  | **Model 2: multinomial (4 groups) ^‡^** | | | | |  | **Model 3: multinomial (3 groups) ^§^** | | |
| --- | --- | --- | --- | --- | --- | --- | --- | --- | --- | --- | --- |
|  | **Consumers vs.**  **Non-consumers** |  | **Low vs. Non** |  | **Medium vs. Non** |  | **High vs. Non** |  | **Medium vs. Low** |  | **High vs. Low** |
|  | **OR (95% CI)** |  | **OR (95% CI)** |  | **OR (95% CI)** |  | **OR (95% CI)** |  | **OR (95% CI)** |  | **OR (95% CI)** |
| **45-54 y *(n=800)*** |  |  |  |  |  |  |  |  |  |  |  |
| **Self-efficacy** | 0.72 (0.43–1.22) |  | 0.98 (0.55–1.73) |  | 0.65 (0.37–1.14) |  | **0.56 (0.32–0.99)^*^** |  | **0.68 (0.47–0.98)^*^** |  | **0.58 (0.41–0.82)^**^** |
| **Perceived benefits** | 1.06 (0.93–1.21) |  | 1.06 (0.92–1.22) |  | 1.09 (0.94–1.26) |  | 0.99 (0.86–1.15) |  | 0.91 (0.82–1.02) |  | 0.94 (0.84–1.06) |
| **Perceived barriers** | **1.44 (1.23–1.68)^***^** |  | **1.28 (1.08–1.51)^**^** |  | **1.46 (1.23–1.73)^***^** |  | **1.74 (1.47–2.07)^***^** |  | **1.12 (1.02–1.24)^**^** |  | **1.32 (1.19–1.45)^***^** |
| **55-64 y *(n=1,021)*** |  |  |  |  |  |  |  |  |  |  |  |
| **Self-efficacy** | 0.67 (0.41–1.08) |  | 0.82 (0.48–1.39) |  | **0.58 (0.34–0.99)^*^** |  | **0.49 (0.29–0.85)^*^** |  | **0.70 (0.49–0.99)^*^** |  | **0.57 (0.40–0.81)^**^** |
| **Perceived benefits** | 1.02 (0.91–1.14) |  | 1.04 (0.92–1.17) |  | 0.95 (0.83–1.09) |  | 0.98 (0.85–1.13) |  | **0.91 (0.83–1.00)^*^** |  | 0.96 (0.86–1.07) |
| **Perceived barriers** | **1.33 (1.17–1.51)^***^** |  | **1.22 (1.07–1.40)^**^** |  | **1.37 (1.19–1.58)^***^** |  | **1.61 (1.39–1.86)^***^** |  | **1.20 (1.09–1.31)^***^** |  | **1.34 (1.21–1.48)^***^** |

Independent variables: self-efficacy, perceived benefits, and perceived barriers. Dependent variable: SSB consumer group. All models were adjusted for sex, education level, perceived health, and body mass index (BMI). SSB, sugar-sweetened beverage; OR, odds ratio; CI, confidence interval. Significance levels: ^*^*p* < 0.05, ^**^*p* < 0.01, ^***^*p* < 0.001.

^1^ Consumer groups were classified based on intake distribution, with non-consumers as the first group (0 times/week), and the remaining consumers divided into tertiles: low consumers (1–2 times/week), medium consumers (3–6 times/week), and high consumers (≥1 time/day).

**^†^ Model 1:** Binary logistic regression comparing SSB consumers vs. non-consumers.

^‡^ **Model 2**: Multinomial logistic regression including all four consumer groups, with non-consumers as the reference group. Model fitting information: *p* < 0.001.

^§^ **Model 3**: Multinomial logistic regression excluding non-consumers, with low consumers as the reference group. Model fitting information: *p* < 0.001.
